# Improving genetic testing pathways for transthyretin amyloidosis in France: challenges and strategies

**DOI:** 10.1186/s13023-024-03370-z

**Published:** 2024-10-29

**Authors:** Bérénice Hebrard, Marie-Lise Babonneau, Philippe Charron, Emilie Consolino, Benjamin Dauriat, Delphine Dupin-Deguine, Dominique Fargeaud, Agnès Farrugia, Anna-Gaëlle Giguet-Valard, Damien Guijarro, Jocelyn Inamo, Julien Jeanneteau, Jean-Michaël Mazzella, Claire-Cécile Michon, Gilles Millat, Frédéric Mouquet, Silvia Oghina, Yann Pereon, Vianney Poinsignon, Julie Pompougnac, Julie Proukhnitzky, Elise Schaefer, Franck Sturtz, Mathilde Trosdorf, Anne Auguste, Giorgia Canali, Alexandre Combes, Benoît Funalot, Thibaud Damy

**Affiliations:** 1 Genetics Department, Henri-Mondor Hospital, AP-HP Henri-Mondor, Créteil, France; 2 Mondor Amyloidosis Network, Créteil, France; 3 Filière Nationale de Santé CARDIOGEN, Paris, France; 4 Association Française Des Conseillers en Génétique, Auriol, France; 5 Genetics and Cardiology Departments, Sorbonne Université, INSERM 1166, Institute of Cardiology and ICAN Institute for Cardiometabolism and Nutrition, Pitié-Salpêtrière Hospital, AP-HP, Sorbonne, Paris, France; 6 Genetics Department, La Timone Hospital, AP-HM, Marseille, France; 7 Medical Genetics Department, Dupuytren Hospital, Universitary-Hospital of Limoges, Limoges, France; 8 Genetics Department, Purpan Hospital, Universitary-Hospital of Toulouse, Toulouse, France; 9 Cardiology Department, Pontchaillou Hospital, Universitary-Hospital of Rennes, Rennes, France; 10 Association Française Contre L’Amylose, Marseille, France; 11 Pierre-Zobda Quitman Hospital, Universitary-Hospital of Martinique, Fort-de-France, France; 12 Filière Nationale de Santé FILNEMUS, Marseille, France; 13 Groupe Hospitalier Mutualiste de Grenoble, Grenoble, France; 14 Unité de Cardiologie Interventionnelle, Institut du Coeur, Clinique Saint-Joseph, Trélazé, Angers, France; 15 UF Cardiogénétique, LBMMS, Hospices Civils de Lyon, Bron, France; 16 UTIC, Hôpital Prive Le Bois, Lille, France; 17 Cardiology Department, Henri-Mondor Hospital, AP-HP Henri-Mondor, Créteil, France; 18 CHU Nantes, Centre de Référence Maladies Neuromusculaires AOC, Euro-NMD, Hôtel-Dieu, Nantes, France; 19 Molecular Genetics, Pharmacogenetic and Hormonology Department, Bicêtre Hospital, AP-HP Paris-Saclay, Kremlin-Bicêtre, France; 20 Medical Genetics Department, Institut de Génétique Médicale d’Alsace, University-Hospital of Strasbourg, Strasbourg, France; 21 Molecular Biology Department, University Hospital of Limoges, Limoges, France; 22 Pfizer, Paris, France; 23 Université Paris Est Créteil, INSERM U955-IMRB, Créteil, France

**Keywords:** ATTR, Genetic testing, Rare disease, Multidisciplinary expert group, Experts’ consensus

## Abstract

Transthyretin amyloidosis (ATTR) is a severe and rare disease characterized by the progressive deposition of misfolded transthyretin proteins, causing irreversible organ damage. Transthyretin amyloidosis can present as a hereditary ATTR or acquired wild-type ATTR form. Genetic testing is critical for determining a hereditary predisposition and subsequently initiating appropriate family screening. In France, strict regulations govern genetic testing that aim to protect patients and their families affected by hereditary diseases such as ATTR. However, challenges persist in establishing an effective genetic testing pathway. A multidisciplinary group of French experts convened to discuss the challenges associated with an ATTR genetic diagnosis and to propose improvement strategies. Key challenges include the lack of pathway standardization, communication gaps between healthcare professionals (HCPs) and patients, and difficulties in complying with regulatory requirements. Concerns about patient data safety and outsourced testing quality further complicate matters. Proposed strategies included the development of stakeholder mapping tools for HCPs and patients, educational programs to improve literacy on genetic testing regulations, increase disease awareness among medical geneticists and genetic counselors, and strengthening HCP-patient communication through educational materials. These initiatives aim to streamline the genetic testing pathway, enhance compliance with regulations, and ultimately provide optimal support for patients and families with ATTR.

## Introduction

Transthyretin amyloidosis (ATTR) is a severe and rare disease characterized by the progressive extracellular deposition of misfolded TTR proteins forming amyloid fibrils and causing irreversible organ damage [1]. Transthyretin amyloidosis is a heterogeneous disease at the genotype and phenotype level. Transthyretin amyloidosis can be hereditary (ATTRv) where the gene bears a pathological variation or acquired wild-type TTR (ATTRwt) where none of the pathogenic variations are found [2, 3]. While patients with ATTRwt predominantly present with cardiac phenotypes (i.e., TTR cardiomyopathy [ATTR-CM]), patients with ATTRv present with various types of phenotypes that can be predominantly neuropathic (i.e., TTR familial amyloid polyneuropathy [ATTR-PN]), cardiac (i.e., ATTR-CM) or mixed [2]. More than 140 disease-causing *TTR* gene variations have been reported [4], with disparities observed in age of clinical onset, organ involvement and prognosis [5].

Between 2011 and 2019, 8950 french patients with incident ATTR-CM were identifed. Incidence rates increased from 0.6/100,000 person-years in 2011 to 3.6/100,000 person-years in 2019 (*p* < 0.001), reaching 2377 new cases in 2019 [6]. A key step for the final diagnosis of ATTR is genetic testing [7]. It is advised that *TTR* genetic testing should be conducted for all patients with an established diagnosis of ATTR to determine if the disease is hereditary (i.e., index patient) and if further family screening is required (i.e., relatives of index cases with no signs or symptoms of the disease) [1, 8–10]. However, to our knowledge, no previously published publication allows us to quantify nationwide genetic testing or genetic counselling in ATTR patients in France.

In France, genetic testing is a highly regulated activity that protects patients and their relatives affected by hereditary diseases, including ATTR, in regards of data privacy, risk of discrimination, right to know or not to know about their condition, and potential psycho-social risks related to misinformation or lack of guidance on how to live with the disease. Thus, genetic testing can only be prescribed by medical geneticists, genetic counsellors (under the direction of a genetic qualified physician) for healthy subjects and, for index cases, also by a non-geneticist physician if familiar with the clinical situation and the familial implications, and if capable of interpreting the results [11, 12]. Prescribers’ obligations include informing the patient of the modalities for relatives’ information, providing written informed consent before the test is performed, delivering the results of the test during a dedicated consultation, and the list of relatives to inform after obtaining the test result [12]. Patients’ obligations are to inform their relatives about the existing genetic results and potential for predictive genetic testing [12]. This legal framework applies to all prescribers and patients in France.

Genetic diseases affect not only the patients with the diagnosed genetic condition or the carrier of the genetic trait, but also parents’ relatives and community in which the patient lives [13]. Patients and their relatives may experience significant emotional distress, anxiety, or depression while learning about and dealing with a rare and debilitating genetic disease that can impact their quality of life [14]. This emotional impact can be increased if the diagnosis announcement occurs in unfavorable circumstances, too rapidly, involving healthcare professionals without adequate training and lack of psychological support for the patient. These risks have prompted discussions among experts regarding the specific challenges related to ATTR. A group of 25 experts comprising eight geneticists, seven cardiologists, four genetic counselors, three psychologists, one neurologist, one nurse and one patient association representative gathered during a 1-day meeting to discuss the current status of the ATTR genetic diagnosis and its challenges, share best practices and propose unique initiatives and strategies to guide a better implementation of ATTR genetic testing in France in order to provide patients with the best care and support. The aim of this article is to describe the key learnings of the expert group’s opinion.

## The challenges

### A challenging homogenization of genetic healthcare journey

**Fig. 1:**
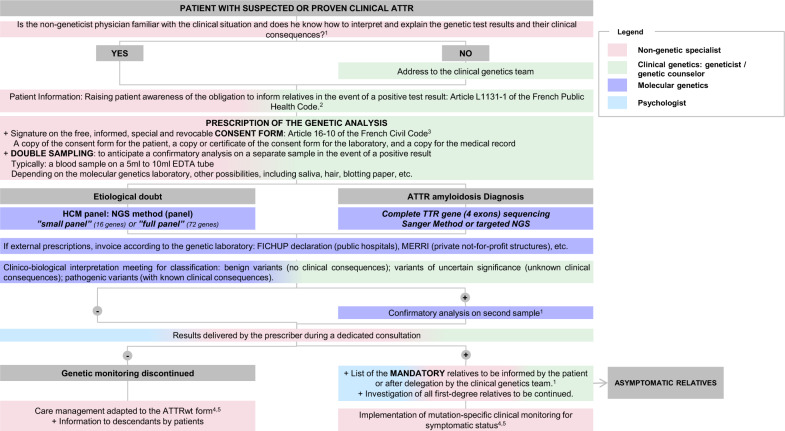
Genetic Pathways for patients with suspected or proven clinical ATTR

**Fig. 2:**
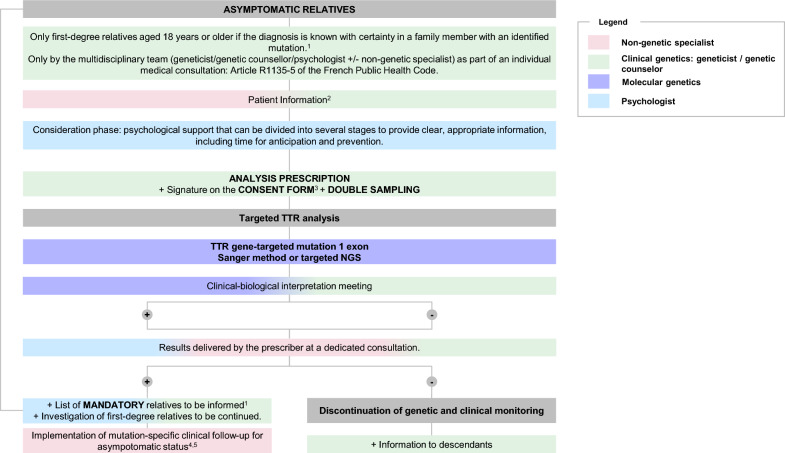
Genetic Pathways for asymptomatic relatives. 1. According to French legislation defining best practices for genetic testing [15]; 2. Defined by the French Public Health Legislation [11]; 3. Defined by the French Civil Code [16]; 4. Filière CARDIOGEN, Centre de référence Amyloses Cardiaques, rotocole National de Diagnostic et de Soins (PNDS) Amyloses Cardiaque (2020–2021); 5. Centre de Référence des Neuropathies Amyloïdes Familiales et Autres Neuropathies Périphériques Rares (NNERf), Protocole National de Diagnostic et de Soins (PNDS) Neuropathie amyloïde familiale

Providing a clear pathway for genetic testing for ATTR patients and their relatives is crucial for optimizing care and outcomes. Experts concluded that there is a high variability in the genetic testing journey of these patients and relatives, stemming from diverse patient profiles and the involvement of numerous HCPs in their management who may not necessarily be ATTR experts. For example, medical geneticists and genetic counselors manage a wide variety of hereditary diseases but few of them are specialists in ATTR. Homogenizing this pathway, such as the ones illustrated in Figs. 1 and 2, would ensure more consistent and efficient information given to the concerned families, the approach to the diagnosis, disease characterization, and ongoing care. Following legally established standardized protocols and guidelines may mitigate the observed disparities, ensure familial screening and specific follow-up adapted to the pathogenic variation identified. This effort to streamline the genetic patient pathway for ATTR, not only aims for better clinical management, but also strives to diminish the inequalities in care experienced by patients and their relatives.

### Need for improvement of the communication between physicians and patients

As ATTRv is a genetic condition, patients’ relatives may be carriers of a gene variation, potentially leading to a severe disease without their knowledge. In this context, the legal framework enforces ATTRv patients to communicate the genetic results to their relatives, to facilitate disease screening, diagnosis and prompt treatment initiation when indicated. All the experts highlighted the challenges associated with ensuring a comprehensive genetic testing protocol for all eligible family members of patients diagnosed with ATTRv. The discussion led to the conclusion that an ineffective communication with the index patient was at the root of the problem. Firstly, HCPs mention the difficulties in conveying the importance of genetic testing for relatives, and thus fail in impending the screening of potential carriers of the gene variation. Secondly, HCPs could be ineffective in communicating patients’ responsibilities related to sharing genetic information and test results to their relatives, as patients themselves are unaware of the implications and significance of their genetic information.

### HCPs’ difficulties in complying with the genetic testing regulations

According to the legal framework on the genetic testing mentioned above, results of tests, even if negative, must be communicated during in-person consultations to provide patients with all the necessary information to understand their results, their implications, their follow-up, care, and treatment, and offer them the possibility to ask questions [18, 19]. Some experts flagged that this directive was not always followed, especially when relatives receive their negative or even positive diagnosis by mail. Experts conveyed that the primary reasons behind this lack of formal consultation were resource limitations. These limited resources, coupled with the time required for laboratories to conduct the tests, can sometimes dramatically lead to lengthy delays up to 1 year. Such delays may lead to situations where patients could be lost to follow-up as they may not have returned to collect their results or even to situations where patients have passed away before obtaining their results and missed the chance of informing their relatives. Furthermore, the experts mentioned that the recent opening of genetic testing performed in private laboratories in France could lead to a surge in the number of tests performed [19]. While the implementation of the private testing pathway, in addition to the public, has the advantage of increasing access to the test, it has the drawback of opening management of ATTR to physicians unaware of regulations for genetic prescription. Hence, there may not be adequate support for these physicians in terms of educational resources for result interpretation, awareness of regulations for genetic prescription, and access to genetic counseling services. This lack of support could result in negative outcomes for both patients and their relatives.

### Patient data safety and quality of genetic testing

The experts’ opinions highlighted the potential risks associated with outsourcing *TTR* sequencing to facilities beyond French borders. First, uncertainties regarding the quality control of the analyses, as certain vendor laboratories lacked specialized expertise in *TTR* sequencing, might be resulting in erroneous results. Second, experts raised that there was lack of data safety guarantee when sending samples abroad—an aspect often overlooked by HCPs due to insufficient awareness of data safety regulation [17].

## Proposed strategies

### Development of stakeholder mapping for HCPs and patients

**Fig. 3:**
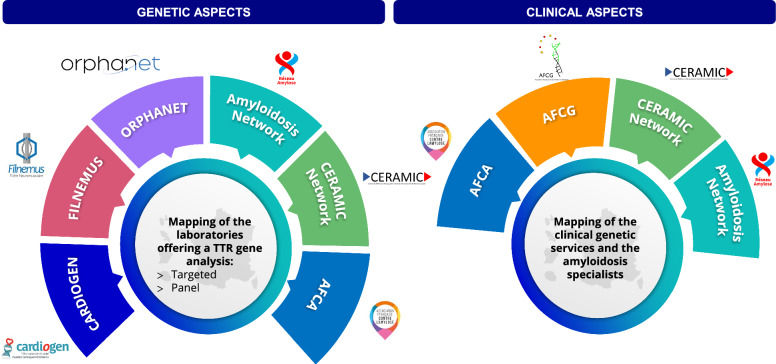
Principles and main stakeholders involved for the mapping of laboratories offering TTR analysis, clinical genetic services and amyloidosis experts

Given that ATTR is a rare and complex disease, HCP expertise on the disease is spread across the territory and hampers families from having rapid access to expert centers for diagnosis and management. Nonetheless, ensuring a consistent implementation of genetic testing across the territory and granting immediate and ATTR expert access to genetic testing is pivotal in delivering high-quality care and to alleviating the loss of opportunity for patients and relatives, as stipulated in the French regulations. This was an important key point that was discussed by the experts, where a broad consensus was reached on the potential implementation of a tool, including specialists mapping across the territory. The mapping should be easily accessible by HCPs and families and should include specialists’ contact information to facilitate referrals or to ask for support and information. The patient associations (e.g., *Association Française Contre l’Amylose* [AFCA] www.amylose.asso.fr), professional associations (*Association Française des Conseillers en Génétique* [AFCG] www.af-cg.fr), and rare disease networks, such as *Réseau Amylose* (www.reseau-amylose.org), *Filière CARDIOGEN* (www.filiere-cardiogen.fr) or *Filière FILNEMUS* (www.filnemus.fr) could have a key role in regularly updating the tool and integrating it in their dissemination channels thereby, patients and relatives are aware of it when seeking support (Fig. 3). However, some logistic aspects should be considered, as centers should agree on the updating of the information and being able to manage the potential cases referred to them.

### Development of educational programs to improve knowledge of the genetic testing regulations

In order to overcome the general lack of awareness of the genetic testing regulations, only a few centers (e.g., Filière CARDIOGEN, CHU Henri Mondor) offer educational programs for genetic testing prescribers [17]. These programs not only aim at improving the awareness of regulatory implications but also the management of ATTR patients. A consensus among experts was reached regarding the need for a broader implementation of such programs in other centers that manage ATTR patients.

Experts agreed on the importance of a pedagogical investment for private practitioners that could include the implementation of medical development programs focusing on the genetic testing regulations and how to communicate to patients their rights and obligations (e.g., obligation to inform relatives, right of not to be informed about the diagnosis) following the example of the already successful program from the CHU Henri Mondor. Additionally, the development of educational materials to be disseminated through the primary amyloidosis stakeholders and networks in France (e.g., AFCA, AFCG, Filière CARDIOGEN, Réseau Amylose) could help increase awareness among private practitioners and facilitate referrals of patients to expert centers.

### Increase disease awareness among medical geneticists and genetic counsellors

Offering an appropriate and personalized ATTR patient health care journey remains difficult because of the variety of clinical profiles, specifically for medical geneticists and genetic counselors who normally have a transversal knowledge of hereditary diseases without a specific specialization in ATTR. The experts decided to formalize educational documents based on different patient profile. These documents would be tailored to genotype–phenotype relation so that users can learn about disease specificities, impact on quality of life and psycho-social implications. Depending on the mutation, disease onset and clinical manifestations will differ. With this tool, medical geneticist and genetic counsellors would be able to provide personalized care to patients and refer them to appropriate organ specialist involved in disease management (e.g. neurologists, cardiologists, or other specialists).

### Strengthen HCP-patient communication

In order to strengthen HCP-patient communication, experts agreed that the development of educational materials, for both HCPs and patients, would be a potential solution. In fact, some hospitals, such as the CHU Grenoble, have already developed documentation for prescribers that could be disseminated to other centers on how to improve communication with patients and their relatives to encourage them to be genotyped. Furthermore, educational materials for patients could be developed with information about the need for genotyping and the potential implications for their relatives, together with links to other sources or patient associations to seek support. Depending on the type of documentation and content, redaction and diffusion would rely on all previously mentioned stakeholders (scientific societies, specialized HCP networks of ATTR or patient association), through all appropriate channels (e-platforms, websites, congresses, etc.). Moreover, language barriers were also identified as potential impediments to effective communication.

### Reinforce patient psychosocial support

Psychosocial support for patients and families could be offered through a dedicated consultation with a clinical psychologist accustomed to genetic counselling processes. Such a consultation could take place both before testing and after the announcement of the diagnosis, with possibilities of short- to medium term follow-up when needed. It could offer emotional support to both the carrier and their relatives and help identify patients at higher mental health risk. In the case of pre-symptomatic consultations, the psychologist supports patients in anticipating different possible diagnostic results, and in such, prepares the diagnosis announcement consultation, and mitigates the risk for relational psychological disorders. These consultations would also contribute to better HCP-patient communication and facilitate extended disease screening and diagnosis in affected families.

## Conclusion

The genetic and phenotypic diversity inherent in ATTR underscores the importance of genetic testing for an accurate diagnosis. The stringent legal framework in France reflects a commitment that ensures ethical standards aimed at protecting patients and their relatives. As legal framework is different depending on the country, local adaptation of patient pathway could be needed, but this approach could be replicable anywhere if every country-specific stakeholder is involved.

Expert opinion delineates significant challenges in the current genetic testing landscape. Disparities in the genetic testing journey, communication of the deficiencies between HCPs and families, and hurdles in regulatory compliance calls for strategic interventions. Moreover, resource constraints leading to excessively long result turnaround times, potential pitfalls associated with outsource testing and concerns regarding data security, were identified as challenges by expert opinion.

To address these intricacies, operational initiatives are proposed, such as the development of a stakeholder mapping tool, educational programs tailored for practitioners, or the creation of informative materials for both HCPs and patients. This paper seeks not only to streamline the genetic testing pathway by aiding in the development of these initiatives, but also to enrich communication, enhance comprehension of the genetic testing regulations, and ultimately provide optimal support and care for patients and their families struggling with the complexities of ATTR.

Finally, in anticipation of future advancements, the next critical phase involves the implementation of the strategic initiatives and the measurement of their effectiveness.

## Data Availability

Not applicable.

## Abbreviations

AFCA: Association Française Contre l’Amylose
AFCG: Association Française des Conseillers en Génétique
ATTR: Transthyretin amyloidosis
ATTR-CM: Transthyretin amyloid cardiomyopathy
ATTR-PN: TTR familial amyloid polyneuropathy
ATTRv: Hereditary ATTR
ATTRwt: Wild-type ATTR
HCP: Healthcare professional
TTR: Transthyretin
